# Differences in F-Wave Characteristics between Spinobulbar Muscular Atrophy and Amyotrophic Lateral Sclerosis

**DOI:** 10.3389/fnagi.2016.00050

**Published:** 2016-03-09

**Authors:** Jia Fang, Liying Cui, Mingsheng Liu, Yuzhou Guan, Xiaoguang Li, Dawei Li, Bo Cui, Dongchao Shen, Qingyun Ding

**Affiliations:** ^1^Department of Neurology, Peking Union Medical College Hospital, Chinese Academy of Medical Sciences, Peking Union Medical CollegeBeijing, China; ^2^Neuroscience Center, Chinese Academy of Medical SciencesBeijing, China

**Keywords:** spinobulbar muscular atrophy, amyotrophic lateral sclerosis, F-wave, giant F-wave, nerve conduction study, motor neuron

## Abstract

There is limited data on the differences in F-wave characteristics between spinobulbar muscular atrophy (SBMA) and lower motor neuron dominant (LMND) amyotrophic lateral sclerosis (ALS). We compared the parameters of F-waves recorded bilaterally from the median, ulnar, tibial, and deep peroneal nerves in 32 SBMA patients, 37 patients with LMND ALS, and 30 normal controls. The maximum F-wave amplitudes, frequencies of giant F-waves, and frequencies of patients with giant F-waves in all nerves examined were significantly higher in the SBMA patients than in the ALS patients and the normal controls. The mean F-wave amplitude, maximum F-wave amplitude, frequency of giant F-waves, and frequency of patients with giant F-waves in the median and deep peroneal nerves were comparable between the ALS patients and normal controls. Giant F-waves were detected in multiple nerves and were often symmetrical in the SBMA patients compared with the ALS patients. The number of nerves with giant F-waves seems to be the most robust variable for differentiation of SBMA from ALS, with an area under the curve of 0.908 (95% CI: 0.835–0.982). A cut-off value of the number of nerves with giant F-waves (≥3) for diagnosing SBMA showed high sensitivity and specificity: 85% sensitivity and 81% specificity vs. ALS patients. No significant correlations were found between the pooled frequency of giant F-waves and disease duration in the SBMA (*r* = 0.162, *P* = 0.418) or ALS groups (*r* = 0.107, *P* = 0.529). Our findings suggested that F-waves might be used to discriminate SBMA from ALS, even at early stages of disease.

## Introduction

X-linked recessive spinobulbar muscular atrophy (SBMA), or Kennedy’s disease, is a relatively benign motor neuron disorder in which life expectancy is only slightly compromised (Chahin et al., 2008). A considerable number of SBMA patients may have been misdiagnosed with other neuromuscular diseases, such as amyotrophic lateral sclerosis (ALS) and progressive muscular atrophy, because a family history is not always available for SBMA patients, and typical symptoms of SBMA can be absent initially and appear later in the disease course (Parboosingh et al., 1997). In some ALS patients, upper motor neuron (UMN) signs appear late in the disease course or not at all (Hama et al., 2012). It is difficult to distinguish SBMA from ALS clinically, particularly when patients lack the classic signs for these diseases. However, it is important to differentiate between SBMA and ALS because these two diseases have different prognosis.

Previous studies have demonstrated differences between SBMA and ALS in sensory action potential amplitudes, needle electromyography manifestations, cortical excitability testing, serum creatine kinase levels, and clinical presentations (Ferrante and Wilbourn, 1997; Hirota et al., 2000; Vucic and Kiernan, 2008; Chahin and Sorenson, 2009; Hama et al., 2012; Jokela et al., 2015). It has been suggested that the frequencies of giant F-waves and repeater F-waves are significantly increased in patients with anterior horn cell disorders compared with the healthy subjects (Petajan, 1985; Ibrahim and el-Abd, 1997). However, differences in the characteristics of F-waves between motor neuron diseases such as SBMA and ALS have not been explored up till now. In particular, the relationship between the duration of motor neuron disease and the frequency of giant F-waves has not been explored. The goal of our study was to compare F-wave characteristics between SBMA and lower motor neuron dominant (LMND) ALS to investigate the clinical value of F-waves for discriminating SBMA from LMND ALS.

## Materials and Methods

### Participants

A total of 32 SBMA patients and 37 LMND ALS patients were recruited consecutively for this study between August 2013 and July 2014. Concentric needle electromyography recordings showed evidence of widespread acute and chronic motor axon loss in both the SBMA and ALS patients. All of the SBMA patients included in this study were clinically and genetically diagnosed with SBMA before entrance into the study (Brooks and Fischbeck, 1995). The age at disease onset was defined for both the SBMA patients and the ALS patients as the age at which the patient first noted motor manifestations, including muscle weakness, atrophy, dysphagia, or dysarthria. For the SBMA patients, gynecomastia, occasional fasciculation, paresthesias, or dysesthesias were not used to determine the disease onset, because these manifestations frequently appear long before the neuromuscular manifestations. Lower motor neuron (LMN) involvement was the predominant finding at examination in all of the ALS patients. All of the ALS patients met the El Escorial World Federation of Neurology criteria for the diagnosis of probable laboratory-supported, probable, or definite ALS during follow-up for 1–2 years. No exaggeration of deep tendon jerks in the limbs of ALS patients could minimize the effect of enhanced monosynaptic reflexes on F-wave amplitude (Ibrahim et al., 1993). We clinically staged the ALS patients using the revised ALS Functional Rating Scale (ALSFRS-R) score (Cedarbaum et al., 1999). Using the Medical Research Council (MRC) score, we assessed the strength of the muscle groups responsible for the following movements: shoulder abduction, elbow flexion and extension, wrist dorsiflexion, finger abduction and thumb abduction, hip flexion, knee extension, and ankle dorsiflexion (all bilaterally), yielding a maximum score of 90 (Menon et al., 2015). Thirty healthy volunteers recruited from the patients’ family members and faculty members served as controls. Since the SBMA patients were all men, all of the ALS patients and the normal controls enrolled in the study were men for consistency. All participants were free of diabetes mellitus, alcohol abuse, other systemic disorders or peripheral neurological diseases.

### Motor Nerve Conduction Study

The electrophysiological study was performed with the subject lying supine in a noise-free room, and with the skin temperature maintained between 32 and 34°C during the experiment. Motor nerve conduction studies were conducted according to the standard belly to tendon technique (Pan et al., 2013). Motor nerve conduction was investigated in the median, ulnar, tibial, and deep peroneal nerves. Recordings were performed with an active electrode placed on the belly of the abductor pollicis brevis (APB), abductor digiti minimi (ADM), abductor halluces brevis (AHB), and extensor digitorum brevis (EDB) bilaterally and with a reference electrode placed 3 cm distal to each active electrode. Stimulation was performed at the wrist (7 cm from the recording electrode), elbow, and axilla for the median nerve; at the wrist (7 cm from the recording electrode), below the elbow, above the elbow, and axilla for the ulnar nerve; at the ankle and popliteal fossa for the tibial nerve; and at the ankle, below the fibular head, and above the fibular head for the peroneal nerve. A ground electrode was placed between the recording and stimulating electrodes. Particular emphasis was paid to exclude the possibility of conduction block by stimulating the tested nerves proximally and comparing the amplitudes of the compound muscle action potential (CMAP) responses evoked at the different stimulus sites. Motor nerve conduction parameters, including distal motor latency (DML) and peak-to-peak CMAP amplitude, were analyzed.

### F-Wave Study

We used the F-wave program installed in a Viking EMG machine with a filter range of 20 Hz–3 KHz, an amplifier gain at 0.5 mV per division, and a sweep speed of 5 ms per division for upper limb nerves and 10 ms per division for lower limb nerves. One hundred consecutive supramaximal stimuli were delivered bilaterally to the median and ulnar nerves at the wrist, tibial nerve and deep peroneal nerve at the ankle with a frequency of 1 Hz and duration of 0.1 ms. The supramaximal intensity was 20% greater than the stimulus intensity that was just sufficient to elicit a maximum amplitude CMAP. According to a previous report, 100 stimuli are recommended to adequately measure F-wave values (Rivner, 2008). Axon reflexes were excluded from the F-wave measurements and were defined as identical late responses with constant latencies occurring in at least 8 of 20 stimuli that frequently preceded and occasionally followed the F-waves (Puksa et al., 2003). Deflections with a peak-to-peak amplitude of 40 μV and higher were accepted as F-waves. The following F-wave variables were recorded: minimum F-wave latency, mean and maximum F-wave amplitude (peak-to-peak), F-wave persistence, frequency of giant F-waves, and frequency of patients with giant F-waves. F-wave persistence was defined as the number of recordable F-waves per 100 stimuli and was expressed as a percentage. An F-wave with an amplitude exceeding the mean value plus 2 standard deviations (SD) of the maximum F-wave amplitudes recorded in normal subjects was considered a giant F-wave (Ibrahim and el-Abd, 1997). There were no significant differences in absolute F-wave amplitudes between the same nerves on the left and right sides in normal controls. The critical amplitude of a giant F-wave was calculated based on the amplitudes of the largest F-waves recorded from the non-dominant side of the healthy controls. In the present study, the calculated critical amplitude for a giant F-wave was 1473.80 μV in the median nerve, 1293.69 μV in the ulnar nerve, 1045.46 μV in the tibial nerve, and 696.93 μV in deep peroneal nerve. This study was approved by the Ethics Committee of Clinical Research of Peking Union Medical College Hospital (Beijing, China) and conformed to the principles of the Declaration of Helsinki. All subjects gave their written informed consent to participate in the study.

### Statistical Analyses

The Kolmogorov–Smirnov test was used to test the normality of the data. The Levene test was used to test the homogeneity of variance between different groups. If the data were normally distributed, a univariate ANOVA was performed for three-group comparisons with a *post hoc* Newman–Keuls test. If data were not normally distributed, a Kruskal–Wallis *H* test with a *post hoc* Mann–Whitney *U* test was used. A χ^2^ test was used for categorical data. The significance level was adjusted using Bonferroni correction for multiple comparisons with α’ < 0.0167. A spearman rank correlation test was conducted to assess the relationship between variables. A *P-*value < 0.05 was considered statistically significant. If not otherwise stated, numbers are expressed as the mean and SD. All of the statistical tests performed were two-sided and were conducted using SPSS for Windows, version 21.0 (SPSS, Inc., Chicago, IL, USA).

## Results

The clinical profiles of the participants are summarized in **Table 1**. The disease duration prior to enrollment in this study was significantly longer in the SBMA patients in comparison with the ALS patients. The mean ALSFRS-R score was 40.30 ± 4.04 (range 30.00–45.00), suggesting a mild-to-moderate level of dysfunction in the ALS cohort. Muscle strength, as measured by the MRC score, was comparable between the SBMA patients and the ALS patients. The CAG repeat size in androgen receptor gene of the SBMA patients in the present study was 49.00 ± 2.49 (range 46.00–53.00).

**Table 1:** Clinical profiles of the participants in the study.

Parameters	SBMA (1)	ALS (2)	Control (3)	*P*-value		
				1 vs. 3	2 vs. 3	1 vs. 2
No.	32	37	30			
Age, year (range)	50.09 ± 9.42 (36.00–70.00)	49.95 ± 8.85 (31.00–69.00)	48.30 ± 10.92 (33.00–73.00)	>0.05	>0.05	>0.05
Height (cm)	171.22 ± 4.98	171.59 ± 5.52	171.70 ± 4.29	>0.05	>0.05	>0.05
Duration, year (range)	8.83 ± 6.90 (2.00–30.00)	1.24 ± 0.81 (0.17–4.00)	NA	NA	NA	**<0.001**
Total MRC score	78.66 ± 8.67 (58.00–90.00)	76.30 ± 9.66 (51.00–90.00)	NA	NA	NA	0.377
Upper limb MRC score	51.19 ± 6.58 (36.00–60.00)	48.65 ± 8.78 (30.00–60.00)	NA	NA	NA	0.241
Lower limb MRC score	27.47 ± 2.87 (22.00–30.00)	28.19 ± 2.86 (21.00–30.00)	NA	NA	NA	0.225

*ALS, amyotrophic lateral sclerosis; SBMA, spinobulbar muscular atrophy; NA, not applicable. Values are mean ± SD. Values with significant differences printed in bold characters. For comparisons of parameters between the SBMA patients, the ALS patients, and the normal controls, Bonferroni adjustment was used and α’ < 0.0167 was considered statistically significant.*

Conduction block or M response temporal dispersion were not found in any of the participants enrolled in the study. The results of nerve conduction studies of the SBMA patients, ALS patients and normal controls are shown in **Table 2**. Satisfactory motor conduction recordings were obtained in all of the SBMA patients that were examined. For the ALS patients, CMAPs were absent in one median nerve, one ulnar nerve, one tibial nerve, and three deep peroneal nerves. When compared with the controls, the CMAP amplitudes were significantly decreased, and the DMLs were significantly prolonged in the median, ulnar, and deep peroneal nerves of both the SBMA and ALS patients. The CMAP amplitudes and DMLs in the tibial nerves were comparable between the SBMA patients and the normal controls. The CMAP amplitudes and DMLs were comparable between the SBMA and ALS patients, except for the significantly decreased CMAP amplitudes in the median nerves of the ALS patients. The APB/ADM CMAP amplitude ratios were significantly reduced in the ALS patients compared with the SBMA patients and the normal controls. The EDB/AHB CMAP amplitude ratios were significantly lower in the ALS patients than in the normal controls, whereas the EDB/AHB CMAP amplitude ratios were similar between the SBMA patients and the normal controls.

**Table 2:** Results of nerve conduction studies.

Parameters	SBMA (1)	ALS (2)	NC (3)	*P*-value		
				1 vs. 3	2 vs. 3	1 vs. 2
**DML (ms)**						
Median nerve	3.23 ± 0.66	3.37 ± 0.62	2.82 ± 0.37	**0.001**	**< 0.001**	0.368
Ulnar nerve	2.32 ± 0.54	2.37 ± 0.38	2.11 ± 0.27	**0.010**	**< 0.001**	0.142
Tibial nerve	3.78 ± 0.74	4.02 ± 0.86	3.60 ± 0.64	>0.05	**< 0.05**	**>0.05**
Peroneal nerve	3.64 ± 0.98	3.84 ± 1.15	3.07 ± 0.66	**0.002**	**< 0.001**	0.214
**CMAP amplitude (mV)**						
Median nerve	10.14 ± 3.85	6.89 ± 4.77	14.31 ± 2.80	**<0.001**	**<0.001**	**<0.001**
Ulnar nerve	11.62 ± 3.13	10.30 ± 4.89	15.95 ± 3.02	**<0.001**	**<0.001**	**0.073**
Tibial nerve	13.33 ± 5.52	12.03 ± 4.61	15.31 ± 4.41	0.025	**0.001**	0.592
Peroneal nerve	5.84 ± 2.54	4.60 ± 2.35	7.42 ± 3.25	**0.015**	**<0.001**	**0.054**
**APB/ADM**	0.91 ± 0.36	0.65 ± 0.32	0.92 ± 0.21	0.962	**<0.001**	**<0.001**
**EDB/AHB**	0.47 ± 0.19	0.41 ± 0.21	0.51 ± 0.21	0.292	**0.009**	0.083

*ADM, abductor digiti minimi; AHB, abductor halluces brevis; ALS, amyotrophic lateral sclerosis; APB, abductor pollicis brevis; CMAP, compound muscle action potential; EDB, extensor digitorum brevis; SBMA, spinobulbar muscular atrophy. Values are mean ± SD. Values with significant differences printed in bold characters. For comparisons of parameters between the SBMA patients, the ALS patients, and the normal controls, Bonferroni adjustment was used and α’ < 0.0167 was considered statistically significant.*

Similar to previous investigations (Peiroglou-Harmoussi et al., 1985), there were no correlations between F-wave amplitudes and age, height, or sex. F-waves were absent in the right deep peroneal nerve in two SBMA patients. For the ALS patients, F-wave responses were absent in one median nerve, one ulnar nerve, two tibial nerves, and six deep peroneal nerves. **Table 3** shows the results of F-wave studies performed in the upper extremity nerves. **Table 4** shows the results of F-wave studies performed in the lower extremity nerves. The minimum F-wave latencies in the median and ulnar nerves of the ALS patients were significantly prolonged compared with the SBMA patients and the normal controls. The minimum F-wave latencies in the tibial and peroneal nerves were comparable between the SBMA patients, the ALS patients and the normal controls. In all nerves studied, the maximum F-wave amplitudes, frequencies of giant F-waves, and frequencies of patients with giant F-waves were significantly increased in the SBMA patients in comparison with the ALS patients and the normal controls. The mean F-wave amplitude, maximum F-wave amplitude, frequency of giant F-waves, and the frequency of patients with giant F-waves in both the median and peroneal nerves were comparable between the ALS patients and the normal controls.

**Table 3:** F-wave studies in upper extremity nerves.

Parameters	SBMA (1)	ALS (2)	Control (3)	*P*-value		
				1 vs. 3	2 vs. 3	1 vs. 2
**Median nerve**						
Min F latency (ms)	24.44 ± 1.72	26.12 ± 3.03	24.72 ± 1.40	0.263	**0.004**	**0.001**
F persistence (%)	50.29 ± 25.77	53.31 ± 33.78	94.85 ± 7.07	**<0.001**	**<0.001**	0.482
Mean Famp (μV)	451.70 ± 266.59	298.13 ± 183.06	302.25 ± 99.21	**<0.001**	0.189	**<0.001**
Maximum Famp (μV)	1523.30 ± 894.59	776.36 ± 491.77	875.32 ± 327.92	**<0.001**	0.040	**<0.001**
Giant F-waves (%)	5.82 ± 12.74	0.30 ± 1.16	0.07 ± 0.33	**<0.001**	0.309	**<0.001**
Frequency of Patients with giant F-waves	25/32	6/37	3/30	**<0.001**	0.730	**<0.001**
**Ulnar nerve**						
Min F latency (ms)	24.38 ± 2.61	25.88 ± 2.32	24.68 ± 1.56	>0.05	**<0.05**	**<0.05**
F persistence (%)	72.07 ± 18.61	75.86 ± 29.63	99.00 ± 2.31	**<0.001**	**<0.001**	**0.006**
Mean Famp (μV)	510.67 ± 539.58	421.75 ± 285.64	305.50 ± 84.94	**<0.001**	**0.015**	0.133
Maximum Famp (μV)	1888.33 ± 1127.67	1109.40 ± 656.90	830.70 ± 279.46	**<0.001**	**0.008**	**<0.001**
Giant F-waves (%)	6.64 ± 10.05	4.81 ± 15.75	0.10 ± 0.48	**<0.001**	**<0.001**	**<0.001**
Frequency of Patients with giant F-waves	28/32	20/37	3/30	**<0.001**	**<0.001**	**0.003**

*ALS, amyotrophic lateral sclerosis; CMAP, compound muscle action potential; Famp, F-wave amplitude; Min F latency, minimum F-wave latency; SBMA, spinobulbar muscular atrophy. Values are mean ± SD. Values with significant differences printed in bold characters. For comparisons of parameters between the SBMA patients, the ALS patients, and the normal controls, Bonferroni adjustment was used and α’ < 0.0167 was considered statistically significant.*

**Table 4:** F-wave studies in lower extremity nerves.

Parameters	SBMA (1)	ALS (2)	Control (3)	*P*-value		
				1 vs. 3	2 vs. 3	1 vs. 2
**Tibial nerve**						
Min F latency (ms)	45.06 ± 5.93	45.43 ± 3.91	44.37 ± 2.98	0.815	0.160	0.305
F persistence (%)	94.28 ± 14.07	95.79 ± 15.65	99.92 ± 0.46	**<0.001**	**0.004**	0.077
Mean Famp (μV)	411.96 ± 195.71	399.49 ± 299.88	271.87 ± 91.93	**0.002**	**0.001**	0.533
Maximum Famp (μV)	1054.07 ± 451.62	798.97 ± 376.37	625.48 ± 172.46	**<0.001**	**0.002**	**<0.001**
Giant F-waves (%)	2.49 ± 4.38	2.05 ± 11.73	0.12 ± 0.14	**<0.001**	**0.003**	**<0.001**
Frequency of Patients with giant F-waves	20/32	12/37	2/30	**<0.001**	**0.010**	**0.013**
**Peroneal nerve**						
Min F latency (ms)	44.25 ± 7.31	44.19 ± 6.00	43.03 ± 3.44	0.624	0.159	0.672
F persistence (%)	33.14 ± 23.92	52.37 ± 25.98	46.93 ± 27.16	**0.007**	0.188	**<0.001**
Mean Famp (μV)	398.88 ± 219.74	227.46 ± 283.16	154.31 ± 91.98	**<0.001**	0.083	**<0.001**
Maximum Famp (μV)	840.35 ± 494.14	470.15 ± 334.20	370.21 ± 202.03	**<0.05**	>0.05	**<0.05**
Giant F-waves (%)	20.46 ± 28.66	5.47 ± 20.24	1.22 ± 7.13	**<0.001**	0.084	**<0.001**
Frequency of Patients with giant F-waves	22/32	10/37	3/30	**<0.001**	0.080	**0.001**

*ALS, amyotrophic lateral sclerosis; CMAP, compound muscle action potential; Famp, F-wave amplitude; Min F latency, minimum F-wave latency; SBMA, spinobulbar muscular atrophy. Values are mean ± SD. Values with significant differences printed in bold characters. For comparisons of parameters between the SBMA patients, the ALS patients, and the normal controls, Bonferroni adjustment was used and α’ < 0.0167 was considered statistically significant.*

The number of nerves with giant F-waves in a patient seems to be the most robust variable for differentiation of SBMA from ALS, with an area under the curve of 0.908 (95% CI: 0.835–0.982), showing “very good” diagnostic utility. A cut-off value of the number of nerves with giant F-waves in a patient (≥3) for diagnosing SBMA yielded high sensitivity and specificity: 85% sensitivity and 81% specificity vs. ALS patients; 93% specificity vs. normal control. The remainder of the F-wave variables had lower diagnostic utility than did the numbers of nerves with giant F-waves in a patient, as shown by lower area under the curve. **Tables 5** and **6** show the diagnostic performance of F-wave parameters in SBMA.

**Table 5:** Diagnostic performance of F-wave parameters in upper extremity nerves in spinobulbar muscular atrophy.

	Vs. ALS				Vs. control			
Parameters	Cut-off value	Sen (%)	Spe (%)	AUC (95% CI)	Cut-off value	Sen (%)	Spe (%)	AUC (95% CI)
**Median nerve**								
Min F latency	25.3 ms	76.7	56.3	0.644 (0.555–0.725) *P* = 0.001	24.1 ms	53.3	73.3	0.609 (0.516–0.697) *P* = 0.039
F persistence	86.0%	93.7	24.7	0.537 (0.450–0.623) *P* = 0.456	83.0%	90.6	91.7	0.968 (0.920–0.991) *P* < 0.001
Mean Famp	269.0 μV	78.1	54.8	0.701 (0.617–0.776) *P* < 0.001	509.0 μV	32.8	100	0.675 (0.580–0.756) *P* < 0.001
Max Famp	1057 μV	70.3	76.7	0.782 (0.705–0.859) *P* < 0.001	1131 μV	65.6	80	0.751 (0.666–0.824) *P* < 0.001
Giant F-wave	1.09%	43.8	93.2	0.686 (0.601–0.720) *P* < 0.0010.001	1.14%	43.7	98.3	0.704 (0.615–0.782) *P* < 0.001
**Ulnar nerve**								
Min F latency	24.8 ms	70.0	69.0	0.701 (0.615–0.778) *P* < 0.001	24.5 ms	63.3	56.7	0.579 (0.486–0.669) *P* = 0.134
F persistence	92%	82.8	54.8	0.637 (0.551–0.718) *P* = 0.005	95.3%	95.3	90.0	0.981 (0.939–0.997) *P* < 0.001
Mean Famp	296 μV	75.0	41.1	0.575 (0.487–0.659) *P* = 0.129	373 μV	57.8	81.7	0.707 (0.618–0.785) *P* < 0.001
Max Famp	1352 μV	64.1	72.6	0.730 (0.648–0.803) *P* < 0.001	1289 μV	65.6	95	0.855 (0.780–0.912) *P* < 0.001
Giant F%	1.03%	65.6	72.6	0.678 (0.593–0.756) *P* < 0.001	1.0%	65.6	96.7	0.815 (0.737–0.879) *P* < 0.001

*ALS, amyotrophic lateral sclerosis; AUC, area under the curve; CI, confidence interval; Max Famp, maximum F-wave amplitude; Min F latency, minimum F-wave latency; Sen, sensitivity; Spe, specificity.*

**Table 6:** Diagnostic performance of F-wave parameters in lower extremity nerves in spinobulbar muscular atrophy.

	Vs. ALS				Vs. control			
Parameters	Cut-off value	Sen (%)	Spe (%)	AUC (95% CI)	Cut-off value	Sen (%)	Spe (%)	AUC (95% CI)
**Tibial nerve**								
Min F latency	44.9 ms	56.9	58.6	0.550 (0.459–0.638) *P* = 0.339	47.8 ms	25.9	89.3	0.510 (0.415–0.605) *P* = 0.858
F persistence	99%	33.3	80.6	0.569 (0.480–0.655) *P* = 0.079	99%	33.3	96.4	0.651 (0.557–0.737) *P* < 0.001
Mean Famp	479 μV	30	89.2	0.533 (0.429–0.635) *P* = 0.581	419 μV	40	89.3	0.669 (0.575–0.753) *P* < 0.001
Max Famp	970 μV	51.7	77.8	0.678 (0.591–0.757) *P* < 0.001	818 μV	68.3	82.1	0.797 (0.712–0.866) *P* < 0.001
Giant F-wave	1.0%	43.3	87.5	0.636 (0.548–0.718) *P* < 0.001	1.0%	43.3	98.2	0.698 (0.606–0.780) *P* < 0.001
**Peroneal nerve**								
Min F latency	38.3 ms	22.0	89.4	0.516 (0.421–0.609) *P* = 0.781	45.5 ms	40.0	86.7	0.533 (0.436–0.629) *P* = 0.585
F persistence	49%	76.9	63.2	0.701 (0.611–0.781) *P* < 0.001	46.0%	75	53.3	0.640 (0.543–0.728) *P* = 0.008
Mean Famp	268 μV	69.2	86.8	0.811 (0.729–0.877) *P* < 0.001	201 μV	82.7	88.3	0.877 (0.802–0.932) *P* < 0.001
Max Famp	586 μV	65.4	76.5	0.742 (0.654–0.818) *P* < 0.001	543 μV	71.2	83.3	0.817 (0.733–0.884) *P* < 0.001
Giant F%	3.1%	51.9	91.2	0.697 (0.606–0.777) *P* < 0.001	1.8%	53.8	98.3	0.750 (0.660–0.827) *P* < 0.001

*ALS, amyotrophic lateral sclerosis; AUC, area under the curve; CI, confidence interval; Max Famp, maximum F-wave amplitude; Min F latency, minimum F-wave latency; Sen, sensitivity; Spe, specificity.*

**FIGURE 1:**
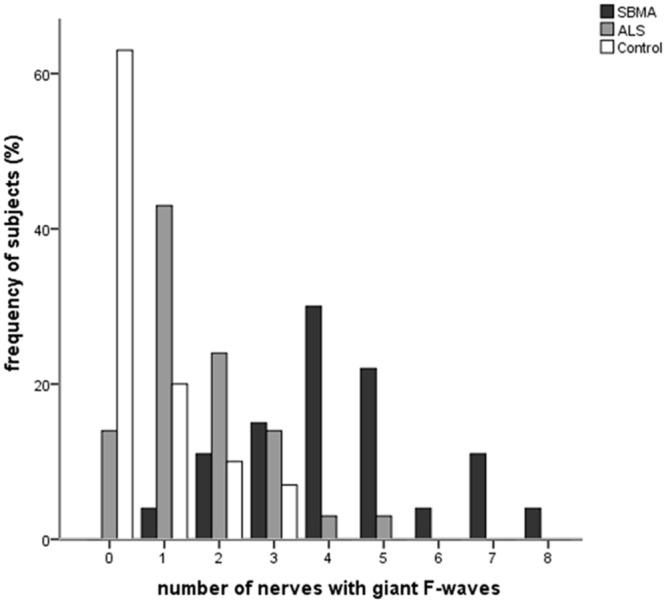
**Histogram of frequencies of subjects exhibiting giant F-waves in different numbers of nerves in the spinobulbar muscular atrophy (SBMA) patients, the amyotrophic lateral sclerosis (ALS) patients, and the normal controls.** Ordinate, frequency of subjects with giant F-waves in different numbers of nerves. Abscissa, number of nerves with giant F-waves.

**FIGURE 2:**
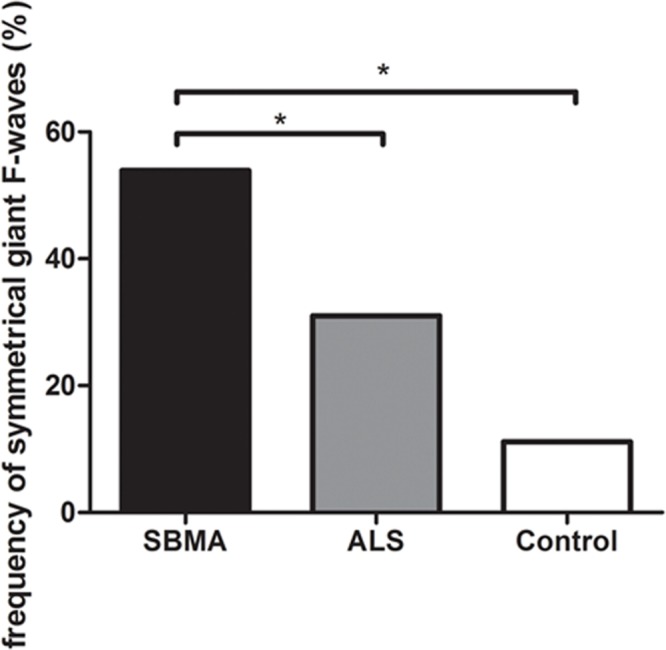
**Histogram of frequencies of giant F-waves recorded symmetrically between the left and right sides in the spinobulbar muscular atrophy (SBMA) patients, the ALS patients, and the normal controls.** Ordinate, frequency of giant F-waves recorded symmetrically between the left and right sides. The frequency of symmetrical giant F-waves between the left and right sides in the SBMA patients was significantly higher than those in the ALS patients (*P* = 0.004) and the normal controls (*P* = 0.001). While the frequencies of symmetrical giant F-waves were comparable between the ALS patients and the normal controls (*P* = 0.171). ^∗^*P* < 0.05.

**Figure 1** shows the frequencies of subjects in each group with giant F-waves recorded in different numbers of nerves. All of the patients with SBMA showed giant F-waves in at least one nerve. Accordingly, a diagnosis of SBMA is questionable if no giant F-waves are recorded. Compared to 56.76% of the ALS patients showing giant F-waves in only one nerve or not at all, 70.37% of the SBMA patients presented with giant F-waves in at least four nerves. The frequencies of patients with giant F-waves in at least three nerves were similar between the ALS patients and the normal controls (*P* = 0.270). Using a cut-off value of six nerves presenting with giant F-waves yielded a sensitivity of 18.25% and a specificity of 100% for differentiating SBMA from ALS. Notably, more than half of the giant F-waves (53.97%) were symmetrical between the left and right sides in the SBMA patients, which was significantly higher than the 31.03% recorded in the ALS patients (*P* = 0.004) and the 11.11% recorded in the normal controls (*P* = 0.001). Giant F-waves typically appeared asymmetrical in the ALS patients, which was similar to the pattern of giant F-waves observed in the normal controls (*P* = 0.171; **Figure 2**).

Correlations between the numbers of giant F-waves and clinical variables were performed. The sum of frequencies of giant F-waves of eight nerves in four limbs was presumed to be an appropriate measure in motor neuron disease characterized by diffuse loss of motoneurons. For the ALS patients, no correlations were found between the pooled frequencies of giant F-waves (22.38 ± 55.37%, range 0–303.00%) and disease durations before electrophysiological examinations (*r* = 0.107, *P* = 0.529), and the severity of the disease as determined by the total MRC scores (*r* = 0.157, *P* = 0.354), or the ALSFRS-R scores (*r* = 0.020, *P* = 0.909). Likewise, there were no correlations between the pooled frequencies of giant F-waves (71.59 ± 47.92%, range 1.49–148.97%) and the disease durations (*r* = 0.162, *P* = 0.418) and the total MRC scores (*r* = -0.052, *P* = 0.796) in the SBMA patients. We divided the SBMA cohort into early (<5 years) versus chronic cases (≥5 years) according to the disease duration. There was no significant difference in the pooled frequencies of giant F-waves between the early cases (54.50 ± 40.68%) and the chronic cases (75.47 ± 49.42%, *P* = 0.314).

## Discussion

The electrodiagnostic features of both the SBMA and ALS patients are consistent with a progressive degeneration of anterior horn cells. These electrodiagnostic features included mildly prolonged DMLs and F-wave latencies, reduced CMAP amplitudes, decreased F-wave persistence, increased F-wave amplitudes, and increased frequencies of giant F-waves, when compared to the normal controls. Compared to the ALS patients, CMAP amplitude abnormalities were less frequent and less pronounced in the SBMA patients. The APB/ADM CMAP amplitude ratio in the ALS patients was reduced significantly compared to that in the normal controls, which is in agreement with the split-hand sign in ALS patients (Eisen and Kuwabara, 2012). However, the APB/ADM CMAP amplitude ratio in the SBMA patients was comparable to that in the normal participants, suggesting a generalized and non-selective process of motoneuron loss. The EDB/AHB CMAP amplitude ratios were significantly decreased in the lower limbs of the ALS patients compared to those in the normal controls, suggesting preferential involvement of motoneurons innervating EDB in ALS. Another possible explanation was that in tibial nerve conduction studies, the AHB CMAP included activity from other foot muscles recorded by the reference electrode, whereas in the CMAP recording of EDB, the contribution by the reference electrode was much smaller (Nandedkar and Barkhaus, 2007; Barkhaus et al., 2011). The possible pathophysiological mechanisms of the extended minimum F-wave latencies in the median and ulnar nerves of ALS patients involve preferential loss of fast conducting fibers, axonal degeneration that slows the propagation of depolarization before conduction is lost entirely, or proximal axonal swellings that slows F-wave turnaround (Carpenter, 1968; Argyriou et al., 2006). While SBMA affects the anterior horn cells diffusely and non-selectively (Ferrante and Wilbourn, 1997; Finsterer, 2010). In ALS patients, 52.70% of the upper limbs were clinically symptomatic, whereas only 31.08% of the lower limbs were clinically affected. This may account for the comparable minimum F-wave latency in the lower extremity nerves between the SBMA patients, the ALS patients and the normal controls. The amplitudes of F-waves are indices of motor unit size and motoneuron excitability (Eisen and Odusote, 1979; McNeil et al., 2013). In anterior horn cell disorders, surviving motor neurons compensate for functional motor unit loss by increasing the size of their motor units through axonal sprouting and reinnervating denervated muscle fibers. The increased mean F-wave amplitudes are probably caused by a higher proportion of large motor units contributing to F-waves (Ibrahim and el-Abd, 1997). The generation of giant F-waves in patients with anterior horn cell disorders may be attributed to axonal sprouting and subsequent reorganization of a muscle’s innervation (Ibrahim and el-Abd, 1997). Other possible mechanisms have been discounted, such as an entire motoneuron pool responding to a stimulus recurrently, an enhanced H-reflex, a repetitive muscle response to an orthodromic motor impulse, and an axon reflex (Ibrahim and el-Abd, 1997). Autopsies showed involvement of the corticospinal tract in 50% to 66% of ALS patients who showed no UMN signs during life (Ince et al., 2003). Consequently, the supraspinal influences on F-waves in patients with LMND ALS cannot be ruled out.

Polyglutamine expansion of the androgen receptor causes SBMA through poorly defined cellular and molecular etiology. High levels of circulating androgens have a role in the pathogenesis of this ligand-dependent neurodegenerative disease (Chua and Lieberman, 2013). Threshold tracking transcranial magnetic stimulation techniques have established normal cortical excitability in SBMA, inferring a lack of significant cortical involvement in this disease (Vucic and Kiernan, 2008). SBMA results from the dysfunction and degeneration of specific motor and sensory neurons (Beitel et al., 2005). The pathogenetic mechanisms of ALS involve accumulation of protein aggregates, oxidative stress, glutamate-induced excitotoxicity, glial dysfunction, neuroinflammation, apoptosis, mitochondrial dysfunction, autophagy, and metal imbalances (Wiedemann et al., 2002; Lederer et al., 2007; Figueroa-Romero et al., 2012). Cortical dysfunction contributed to the pathogenesis of ALS (Menon et al., 2014). Glutamate-mediated cortical hyperexcitability may underlie the different rate of degeneration of motoneurons in ALS (Menon et al., 2014). Compared with ALS, SBMA typically has a relatively slower progression. The differences in F-wave characteristics between the SBMA and ALS patients may be attributed to the distinct pathogenesis and the competing effects of degeneration and regeneration within the motor unit. When a muscle is partly denervated, the muscle fibers that have retained innervation may hypertrophy, and collateral reinnervation may take place (McComas et al., 1971). An increased number of markedly hypertrophic fibers were consistently observed in SBMA patients, whereas this feature is not common in ALS patients (Chahin and Sorenson, 2009). Motor neurons are lost as motor neuron disease progresses, and this can be demonstrated by neurophysiological measures that include CMAP amplitudes, motor unit estimates, and motor unit number indices (Neuwirth et al., 2010). Given the large difference in disease duration between SBMA and ALS, the relative paucity of giant F-waves in ALS likely reflects a decreased efficiency of the burden of reinnervation in surviving motoneurons. Therefore, the diagnosis of SBMA should be questioned if no giant F-waves are recorded. The significantly increased frequency of giant F-waves in SBMA suggests that an increased number of giant F-waves may indicate a decreased rate of the degenerative process. Although electromyography detected diffused neurogenic lesion in both the SBMA and ALS patients. Compared with the ALS patients, giant F-waves were liable to appear in more nerves and symmetrically in SBMA patients. In SBMA, the LMNs are affected diffusely and non-selectively, whereas in ALS, the rate of motor unit loss differs according to the nerve and muscle, as well as the site of disease onset (Baumann et al., 2012). The present study demonstrated that the number of nerves with giant F-waves in a patient was the best index to diagnose SBMA among the multiple F-wave parameters. In our study, the frequencies of giant F-waves were not correlated with disease duration. In addition, the pooled frequency of giant F-waves was similar between the chronic SBMA patients and the early SBMA patients. In other words, this finding supported the clinical utility of giant F-waves in discriminating SBMA from ALS even in the early disease course. Although the SBMA patients had a long duration from disease onset, this did not seem to affect our analyses.

Of note, the mean F-wave amplitude, maximum F-wave amplitude, frequency of giant F-waves, and the frequency of patients with giant F-waves in the median and deep peroneal nerves were comparable between the ALS patients and normal controls. This similarity may be related to the differential involvement of the upper and lower limb muscles in ALS patients (Menon et al., 2013b; Simon et al., 2015). In ALS, there are disparities in muscle activation during physical activity, cortical modulation, or local spinal modulation between different muscle pairs, such as APB and flexor pollicis longus, APB and ADM, or tibialis anterior and soleus (Menon et al., 2013a, 2014; Shibuya et al., 2013; Simon et al., 2015). Previous studies of ALS demonstrated that LMN degeneration in APB or EDB is faster than that in ADM (Kuwabara et al., 1999; Baumann et al., 2012). The dissociated involvement of the small hand muscles in which APB motoneurons are more severely affected than ADM motoneurons, which is termed the “split-hand” syndrome, reflects part of pathophysiology in ALS (Kuwabara et al., 1999). In the present study, preferential involvement of APB and EDB in ALS was indicated.

There are several limitations to our study. The occurrence of giant F-waves has been demonstrated in other anterior horn cell diseases (Ibrahim and el-Abd, 1997). Therefore, disease control group including patients with other LMN syndrome, such as adult onset spinal muscular atrophy, or patients with sequelae following paralytic poliomyelitis, could be included in further study. The number of SBMA and ALS patients included in the present study was too small to draw a generalizable conclusion. Further studies with larger sample sizes are necessary to validate our present findings. In addition, muscle weakness in SBMA patients is usually more severe in the proximal muscles (Ferrante and Wilbourn, 1997). Accordingly, F-waves recorded from small muscles in the distal extremities cannot thoroughly reflect the pathological changes in SBMA. This study was based on a series of 100 F-waves in each nerve, which resulted in a large number of stimuli that were delivered in a single subject. Further study is needed to determine an adequate sample size of F-waves for group comparisons of giant F-waves. Another potential limitation of the study was that the upper range of disease duration in the ALS patients was 4 years. Giant F-waves may be more common in ALS patients with a longer disease duration and it is difficult to differentiate ALS patients with a long duration from SBMA patients. Therefore, additional studies that include ALS patients with longer disease durations are needed. Finally, most SBMA patients showed a longer CAG repeat (≥47), while only two SBMA patients showed a shorter CAG repeat (<47) in the present study. This was consistent with a motor-dominant phenotype of the SBMA patients in our study, such as clinical symptoms of muscle weakness and atrophy, decreased CMAP values, and evidence of widespread acute and chronic motor axon loss in electromyography (Suzuki et al., 2008). Further studies enrolling larger numbers of SBMA patients particularly SBMA patients with a shorter CAG repeat (<47) are needed to investigate the value of F-waves in distinguishing two subgroups of SBMA patients.

Based on the findings of the present study, a significant increase in the frequency of giant F-waves, especially in the median and deep peroneal nerves, or giant F-waves recorded from multiple nerves (≥3) or symmetrically between the left and right sides in male patients with LMN syndromes, are highly suggestive of SBMA. In these cases, genetic testing is recommended, even when the typical clinical features of SBMA are absent.

## Author Contributions

Conceived and designed the experiments: JF, LC; performed the experiments: JF, LC; analyzed the data: JF, LC, ML, YG; contributed reagents/materials/analysis tools: ML, YG, XL, DL, BC, DS, QD; contributed to the writing of the manuscript: JF, LC.

## Conflict of Interest Statement

The authors declare that the research was conducted in the absence of any commercial or financial relationships that could be construed as a potential conflict of interest.
